# Contributions of Latin American researchers in the understanding of the novel coronavirus outbreak: a literature review

**DOI:** 10.7717/peerj.9332

**Published:** 2020-06-04

**Authors:** Karen Y. Fiesco-Sepúlveda, Luis Miguel Serrano-Bermúdez

**Affiliations:** 1 Epidemiology Program, Faculty of Health Sciences, Universidad Surcolombiana, Neiva, Colombia; 2 Bioprocesses and Bioprospecting Group, Universidad Nacional de Colombia, Bogotá D.C., Colombia

**Keywords:** COVID-19, SARS-CoV-2, Pandemic, Severe acute respiratory syndrome, Coronavirus disease

## Abstract

This article aimed to give the visibility of Latin American researchers’ contributions to the comprehension of COVID-19; our method was a literature review. Currently, the world is facing a health and socioeconomic crisis caused by the novel coronavirus, SARS-CoV-2, and its disease COVID-19. Therefore, in less than 4 months, researchers have published a significant number of articles related to this novel virus. For instance, a search focused on the Scopus database on 10 April 2020, showed 1,224 documents published by authors with 1,797 affiliations from 80 countries. A total of 25.4%, 24.0% and 12.6% of these national affiliations were from China, Europe and the USA, respectively, making these regions leaders in COVID-19 research. In the case of Latin America, on 10 April 2020, we searched different databases, such as Scopus, PubMed and Web of Science, finding that the contribution of this region was 2.7 ± 0.6% of the total publications found. In other words, we found 153 publications related to COVID-19 with at least one Latin American researcher. We summarized and processed the information from these 153 publications, finding active participation in topics like medical, social and environmental considerations, bioinformatics and epidemiology.

## Introduction

Severe acute respiratory syndrome coronavirus 2 (SARS-CoV-2) is a novel virus that mainly affects the respiratory system through the new coronavirus disease 2019 (COVID-19) (Ciotti et al., 2020). COVID-19 has spread quickly; thus, on 11 March 2020, the World Health Organization (WHO) declared it as a pandemic (Hussain, Bhowmik & Do Vale Moreira, 2020). Since the first cases reported in Wuhan, China, in December 2019, until 20 April 2020, SARS-CoV-2 has affected most countries in the world, with nearly 2.5 million people infected and 170,000 deaths (Johns Hopkins University, 2020). Latin America is no stranger to this reality, totaling approximately 100,000 cases and 5,500 deaths, as shown in Table 1 (Johns Hopkins University, 2020). However, these data may be lower than actual numbers because the number of tests per million inhabitants remains low, which is caused by factors such as the limited availability of tests and the difficulty of monitoring people without facilities like indigenous populations, vulnerable groups and Venezuelan refugees (Oliveira, Abranches & Lana, 2020; Torres & Sacoto, 2020).

**Table 1:** Summary of the COVID-19 outbreak affectation in the Latin American countries. Total cases and total deaths are reported until 20 April 2020 (Johns Hopkins University, 2020).

Country	First case	Cases	Deaths
Argentina	Mar 3	2,941	136
Bolivia	Mar 10	564	33
Brazil	Feb 25	40,581	2,845
Chile	Mar 3	10,507	139
Colombia	Mar 6	3,963	189
Costa Rica	Mar 6	662	6
Cuba	Mar 11	1,087	36
Dominican Republic	Mar 1	4,964	235
Ecuador	Feb 14	10,128	507
El Salvador	Mar 18	218	7
Guatemala	Mar 13	289	7
Haiti	Mar 2	47	3
Honduras	Mar 11	477	46
Mexico	Feb 27	8,261	686
Nicaragua	Mar 18	10	2
Panama	Mar 8	4,467	126
Paraguay	Mar 7	208	8
Peru	Mar 6	16,325	445
Uruguay	Mar 13	528	10
Venezuela	Mar 13	256	9
Total		106,483	5,475

SARS-CoV-2, as SARS and MERS, belongs to the family *Coronaviridae*, has a zoonotic origin, and can remain on some surfaces for considerable periods (Ciotti et al., 2020; Van Doremalen et al., 2020). Additionally, COVID-19 is a new disease with no yet vaccines or targeted drugs, making the containment of the outbreak difficult (Carnero Contentti & Correa, 2020). Therefore, the recommendation is the self-isolation to reduce COVID-19 spreading, especially in more susceptible people as older adults or patients with comorbidities (Diaz-Quijano, Rodriguez-Morales & Waldman, 2020). More general aspects of the current outbreak have been published in review articles according to available information in the moment of publication. These reviews include other zoonotic diseases (such as SARS and MERS), outbreak chronology, virus characteristics, zoonotic links, transmission, diagnosis, disease characteristics, therapeutics and treatments, prevention, epidemiological surveillance and control (Ciotti et al., 2020; Cupertino et al., 2020; Huang et al., 2020; Millán-Oñate et al., 2020; Palacios Cruz et al., 2020; Rodriguez-Morales et al., 2020a; Sifuentes-Rodriguez & Palacios-Reyes, 2020; Siordia, 2020; Wu et al., 2020; Zhu et al., 2020).

In the past, during SARS and MERS outbreaks, research focused on coronaviruses increased significantly, which was led by researchers from China and the USA (Bonilla-Aldana et al., 2020b). This new outbreak is not an exception because thousands of articles have been published in less than four months, where China, Europe and the USA are leaders in the number of publications. In the case of Latin America, it is a region with an increasingly high contribution to science; thus, our question was, what are the contributions of Latin American researchers in understanding this novel coronavirus outbreak? Therefore, our purpose in this review was to highlight the contributions of this region in the comprehension of SARS-CoV-2 and COVID-19. The literature survey consisted of revising and summarizing publications with Latin American researchers. Keeping in mind that several researchers from this region work together with researchers from other continents, we included publications submitted by these types of international research groups. Hence, the relevance of this review focused on finding the research interests of Latin American researchers according to global and regional priorities.

## Survey Methodology

### Search strategy

We performed the present review following the PRISMA guidelines. The search was done on 10 April 2020, using Scopus, Web of Science, PubMed, ScienceDirect, Wiley, SAGE, LILACS and SciELO databases because they are the main academic literature collections globally and regionally. Other databases like Springer Link were excluded because they do not allow to filtrate by affiliation. The search equation used had (“COVID 19” OR “COVID-19” OR “SARS-CoV-2” OR “SARS CoV 2” OR “SARS-CoV 2” OR “2019-nCoV” OR “2019 nCoV” OR “nCoV-2019” OR “nCoV 2019” OR “hCoV-19” OR “hCoV 19”) in all fields and (Argentina OR Bolivia OR Brasil OR Brazil OR Chile OR Colombia OR Cuba OR Ecuador OR Salvador OR Guatemala OR Haiti OR Honduras OR Mexico OR Nicaragua OR Panama OR Paraguay OR Peru OR Dominicana OR Uruguay OR Venezuela) in affiliation field. We did not consider preprints during the search stage. No interfaces were used in the present literature review.

### Article selection and data extraction

After the search stage, both reviewers (KYFS and LMSB) removed all duplicated publications, which included a manual revision because some publications were simultaneously in English, Spanish, or Portuguese. Later, we performed a second manual revision to verify that all publications had at least one researcher with a Latin American affiliation. After these two manual revisions, we did not exclude more publications, and final publications were included in the qualitative synthesis. Before the qualitative synthesis, we collected the following information, which was used in the bibliometric analysis: title, authors, journal, DOI, type of publication, national affiliation of Latin American researchers, and topic of publication.

### Data analysis

We summarized information from the collected publications according to the type of publication, the topic of publication and the national affiliation in the “Bibliometric Analysis” section. The first purpose of this section was to quantify contributions of the region in the global context, and the contribution by country in the regional context. The second purpose was to classify the publications by topic and type, which allowed us to organize the next sections of this literature review. In the following two sections, “Phylogenetic and Molecular Understanding” and “Medical Contributions”, we compiled information from research articles and reviews. The last section, called “Additional Concerns”, was included to highlight contributions not covered in the two main topics, but discussed in the remaining publications (commentaries, letters to the editor, editorials, communications, perspectives, points of view and contributions).

## Bibliometric Analysis

**Figure 1:**
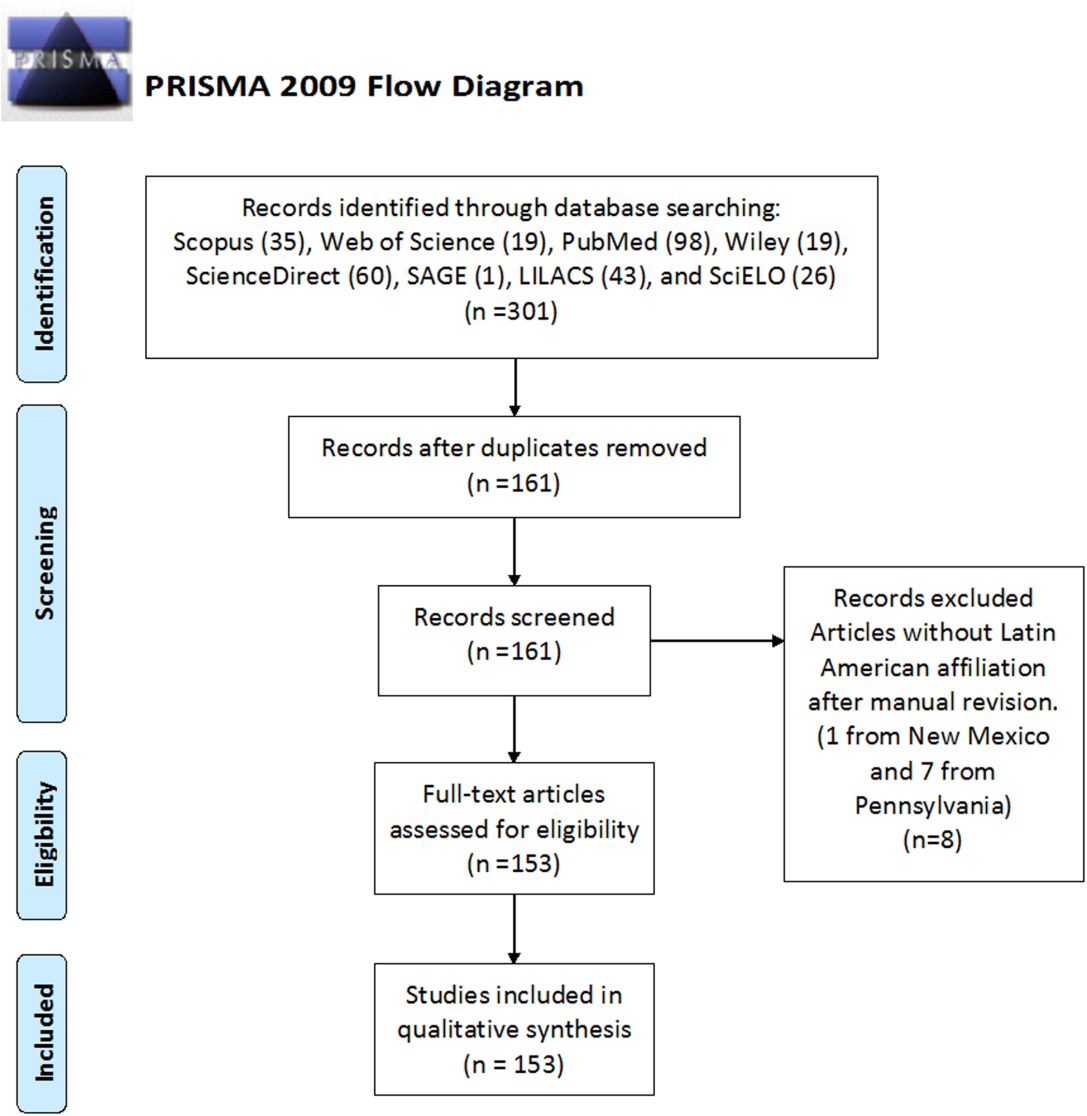
PRISMA Flow diagram of selection process of COVID-19 or SAR-CoV-2 publications containing researchers with Latin American affiliation. Identification stage was performed on 10 April 2020.

**Figure 2:**
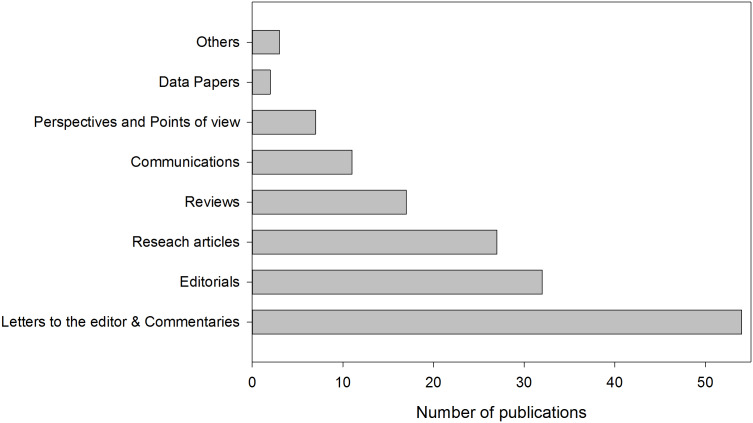
Classification of publications by category. Other publications refer to: consensus statement, contribution and technical note.

Following the PRISMA guidelines shown in Fig. 1, we found 301 publications in the considered databases; this number decreased to 161 after excluding duplicates. Later, we manually excluded eight additional publications due to affiliations from New Mexico (1) and Pennsylvania (7) were confused with Mexico and Panama, respectively. Therefore, this qualitative analysis included 153 publications, which contained at least one researcher with Latin American affiliation (see File S1 for complete information of publications). We highlight that several publications were not exclusively submitted by Latin American researchers, some of which are part of research groups together with North American, European, or Asian researchers. Figure 2 presents the classification of publications by type, where most of them were letters to the editor or commentaries, editorials and research articles.

**Figure 3:**
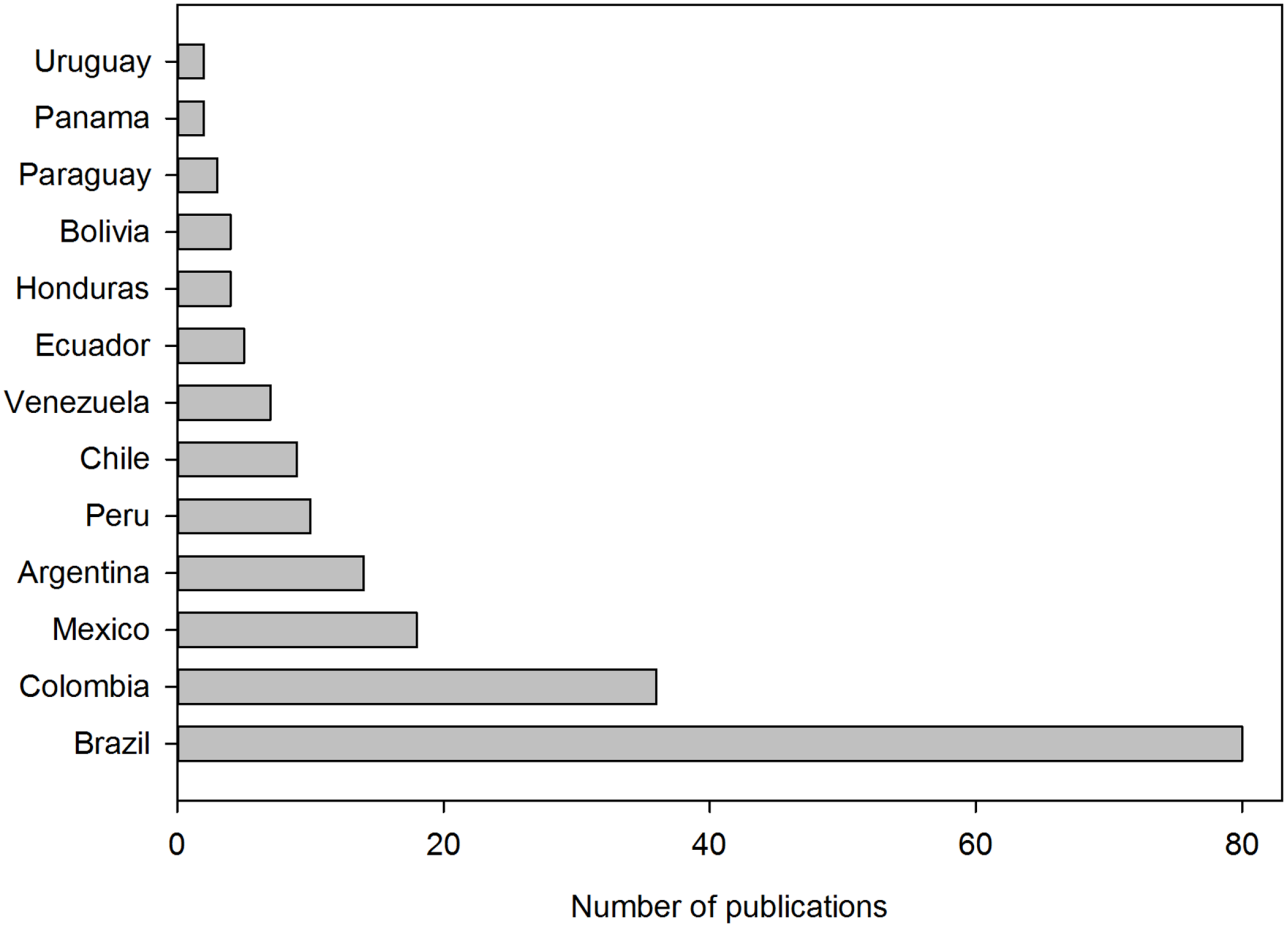
Classification of publications according to the national affiliation. Several authors from the same country in a publication were counted as one contribution to the country. Publications with authors from different countries were counted as one for each country. Latin American countries not shown had no publications until 10 April 2020.

Regardless of the number of authors from the same country but different institutions, among all publications, we highlight that 15 of them were submitted by groups in which there were researchers from at least two Latin American countries. Therefore, these publications were counted for each nation involved; thus, these 15 publications have 56 national affiliations. Most of these publications were due to the Latin American Network of Coronavirus Disease 2019-COVID-19 Research (LANCOVID-19), which was created to integrate the region around this new outbreak (Rodriguez-Morales et al., 2020c). The remaining 138 publications were submitted by groups in which there were researchers from a single Latin American country. Therefore, these publications were counted once for the country, disregarding whether they were submitted by one or more researchers with the same national affiliation; in other words, these 138 publications have 138 national affiliations. In summary, the 153 publications accounted for 194 national affiliations. Figure 3 shows publications by national affiliation, where Brazil had the highest contribution with 80 publications, followed by Colombia, Mexico and Argentina, with 36, 18 and 14 publications, respectively. Conversely, the following Latin American countries had no publications: Cuba, Costa Rica, Dominican Republic, El Salvador, Guatemala, Haiti and Nicaragua.

We did the same search without the affiliation field restriction. We found 1,224, 615, 3,538, 1,841, 665, 48, 2,627 and 34 publications in Scopus, Web of Science, PubMed, ScienceDirect, Wiley, SAGE, LILACS and SciELO databases, respectively. Hence, publications with Latin American researchers in these databases, Fig. 1, represent 2.9%, 3.1%, 2.8%, 3.3%, 2.9%, 2.1%, 1.6% and 76.5% of all publications, respectively. Excluding SciELO, which is a Latin American database, the contribution of the region was 2.7 ± 0.6%. This low value could be associated with the science gap (gap in science funding, technology, facilities) between the region and the developed countries. However, other possibilities are the late coronavirus appearance in the region (between February and March), as opposed to the initial outbreak (December 2019) and the number of Latin American cases (nearly 4% world total), as shown in Table 1.

**Figure 4:**
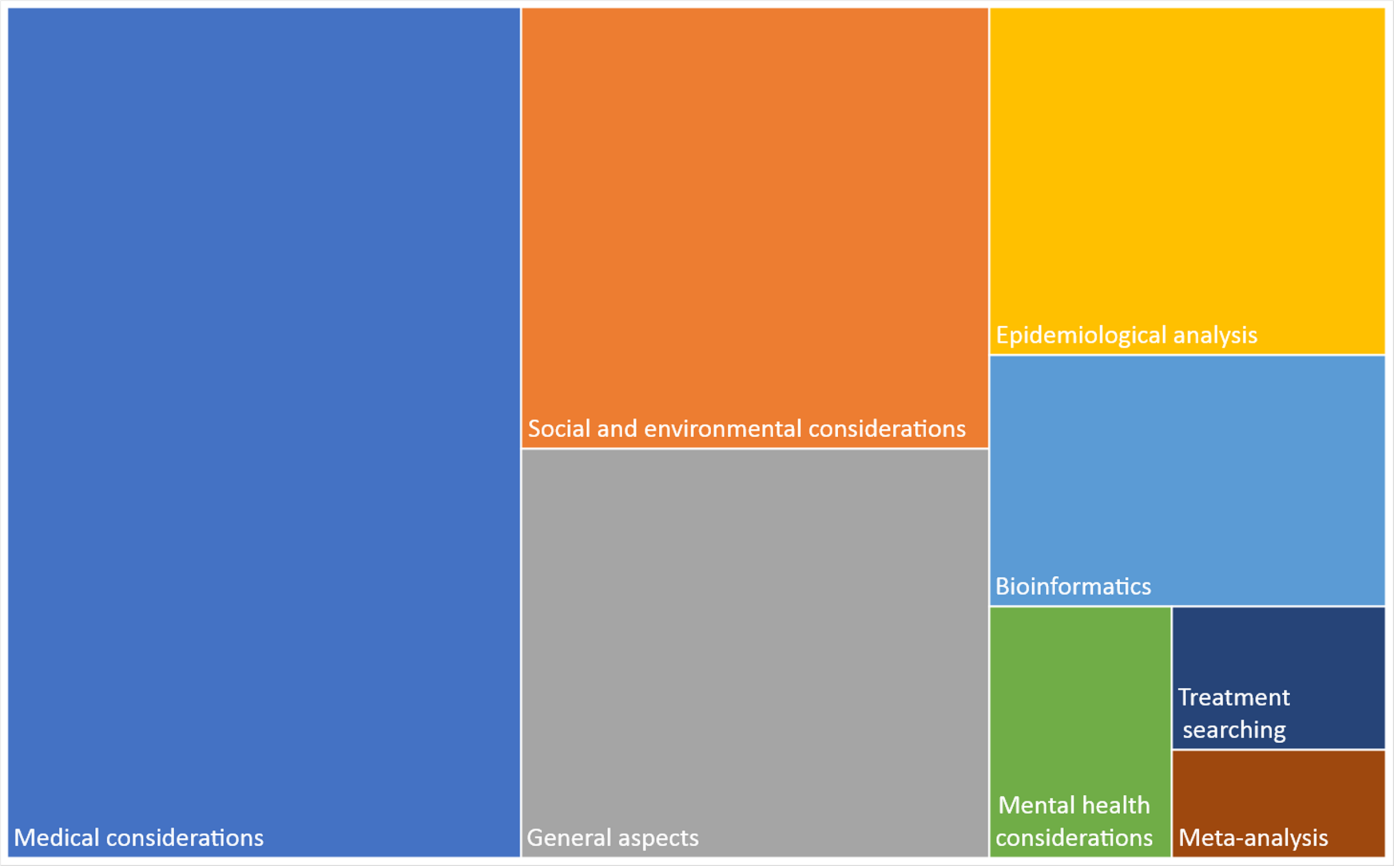
Classification of publications by topic. Medical considerations refer to surgery recommendations, diagnosis, comorbidities, medical guidelines, dentistry considerations, among others. The general aspects include zoonotic links, spreading, origin, disease, surveillance, among others. Bioinformatics refers to molecular and phylogenetic analyses, molecular simulations, genetic annotations, among others.

Finally, Fig. 4 shows the classification of publications by topic, which were medical considerations (surgery recommendations, diagnosis, comorbidities, medical guidelines, dentistry considerations, among others), social and environmental considerations, general aspects (zoonotic links, spreading, origin, disease, surveillance, among others), epidemiological analyses, bioinformatics (molecular and phylogenetic analyses, molecular simulations, genetic annotations, among others), mental health considerations, search for potential treatments, and meta-analyses. Excluding the general aspects, the remaining topics are shown in the following sections.

## Phylogenetic and Molecular Understanding

The researcher with the highest number of research articles was Ph.D. Marta Giovanetti, who has contributed to SARS-CoV-2 understanding through bioinformatic analyses (Angeletti et al., 2020; Benvenuto et al., 2020a, 2020b, 2020c, 2020d; Cleemput et al., 2020; Giovanetti et al., 2020a, 2020b). Her first research focused on a phylogenetic analysis of SARS-CoV-2, finding that among the viruses compared, this virus is closely related to bat-SL-CoVZXC21 (GenBank ID MG772934.1), while the least related is MERS (Benvenuto et al., 2020b). However, Cárdenas‐Conejo et al. (2020) proposed that SARS-CoV-2 has a closer relation to bat-SL-CoV-RaTG13 (GenBank ID MN996532.1), then, authors suggested first that SARS-CoV-2 is unlikely to come directly from pangolin viruses, and second, that if SARS-CoV-2 has a recombinant origin, this recombination did not happen in ORF1ab. Nevertheless, both Benvenuto et al. (2020b) and Cárdenas‐Conejo et al. (2020) concluded that the novel virus could come from a bat SARS-like coronavirus isolate, which is in agreement with reports from the GISAID database (Hadfield et al., 2018; Sagulenko, Puller & Neher, 2018).

Subsequent studies of the Giovanetti group found differences in the superficial spike protein S of SARS-CoV-2 through structural analyses, which could give a higher ability to infect humans when compared to other coronaviruses (Benvenuto et al., 2020b). This ability could be attributed to two mutations found in the non-structural protein 2 (nsp2) and nsp3, both originating from a possible positive pressure (Angeletti et al., 2020). Similarly, evaluating in silico molecular interactions between the human angiotensin-converting enzyme 2 (ACE2) receptor and the spike protein of some coronaviruses, Ortega et al. (2020a) found that SARS-CoV-2 has some modified residues. Such residues could improve the recognition and interaction with the ACE2 receptor, providing SARS-CoV-2 with a higher infectiousness, which is in agreement with another study published simultaneously (Andersen et al., 2020). Likewise, performing in silico molecular interactions, Ortega et al. (2020b) evaluated the interaction between the protease of SARS-CoV-2 and some protease inhibitors as a strategy to control COVID-19 infection. The most energetic interactions predicted were using Saquinavir, Lopinavir, and Tipranavir, which are treatments for HIV patients; however, experimentation is required to validate these simulations.

In a later study, the Giovanetti group analyzed SARS-CoV-2 mutations through time, finding two variations located in nsp6 and ORF10, which could be caused by a positive selective pressure, leading to a lower protein structure stability and possibly (awaiting for evidence) a higher virulence (Benvenuto et al., 2020a). Cárdenas‐Conejo et al. (2020) similarly detected variations in nsp6 and eight deleted amino acids in nsp1 from some Japanese virus strains. Although these in silico studies are a first approach and require experimental validation (Ciccozzi et al., 2020), they could also be a first step to aid in identifying treatments or vaccines.

Lastly, Giovanetti simultaneously contributed to another research group to develop and validate an open-access tool, called the Genome Detective Coronavirus Typing Tool, which analyzes SARS-CoV-2 genomes to generate new knowledge of COVID-19 outbreak (Cleemput et al., 2020).

**Figure 5:**
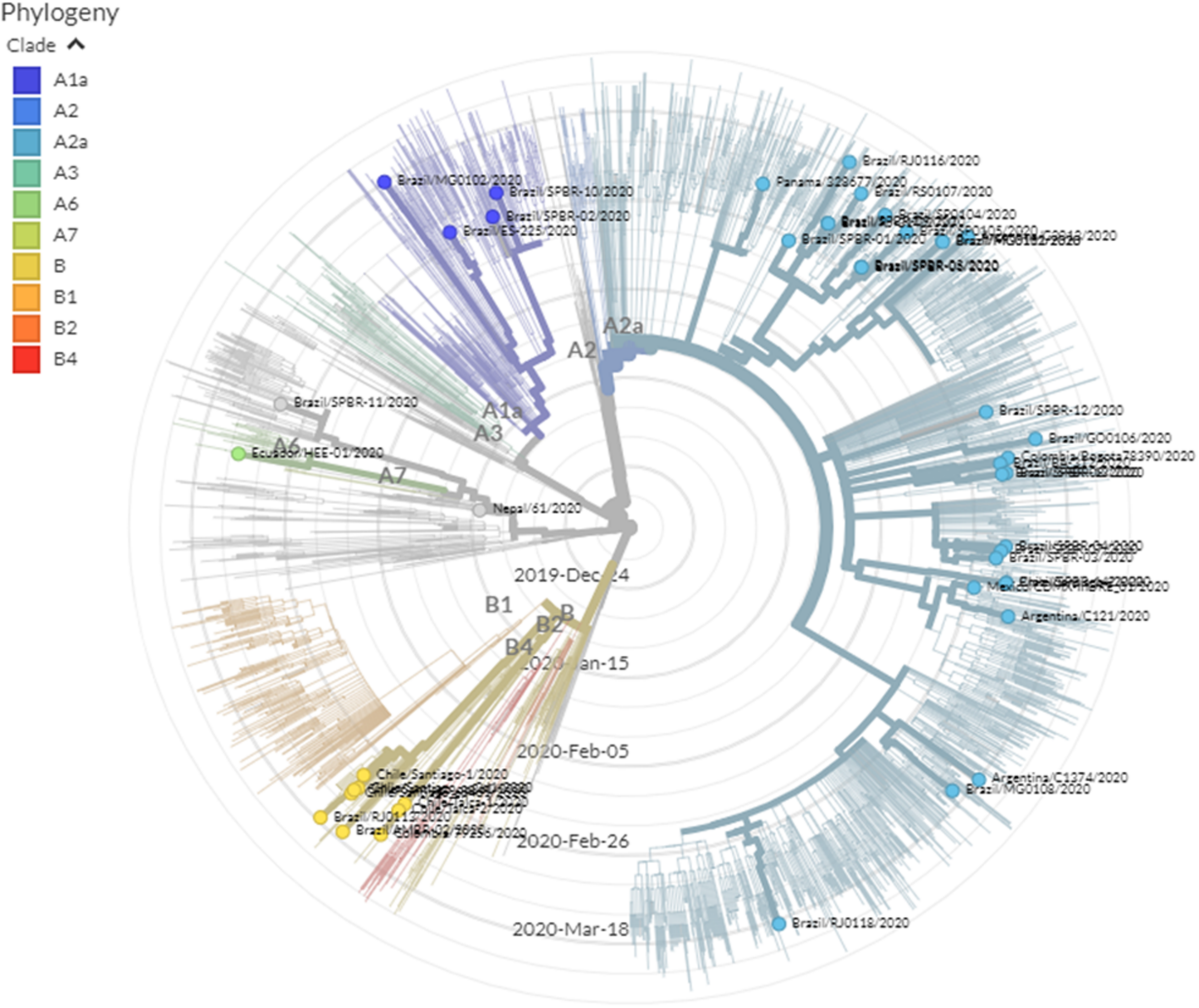
Phylogenetic location of genome sequences of some strains isolated in Latin America in the SARS-CoV-2 phylogenetic tree. The phylogenetic tree was generated and adapted from the GISAID database (Hadfield et al., 2018; Sagulenko, Puller & Neher, 2018). The tree was retrieved on 20 April 2020.

Concerning the sequencing of SARS-CoV-2 genomes to understand this novel coronavirus, some Latin American researchers have contributed to the publication of sequences of isolated strains from countries such as Chile (Castillo et al., 2020) or Nepal (Sah et al., 2020). Researchers from other countries have also sequenced the genomes of strains from Argentina, Brazil, Chile, Colombia, Ecuador, Mexico, Panama, Peru and Uruguay, totaling 98 genome sequences until 20 April 2020 (see File S2 for detailed information of all sequences). The GISAID database has these 98 sequences collected along with 10,380 others, meaning that Latin American contribution is near 0.94% (Hadfield et al., 2018; Sagulenko, Puller & Neher, 2018). Table 2 summarizes this information by country, showing that Brazil and Mexico have the highest number of sequenced genomes, 52 and 17, respectively. Figure 5 presents some of the Latin American SARS-CoV-2 strains in the phylogenetic tree, evidencing the high heterogeneity among them because they belong to different clades.

**Table 2:** Summary of genome sequences of SARS-CoV-2 strains isolated in Latin America and collected in GISAID database. Information updated on 20 April 2020 (Hadfield et al., 2018; Sagulenko, Puller & Neher, 2018).

Country	Submitting lab	Location	Total
Argentina	Instituto Nacional Enfermedades Infecciosas C.G.Malbran	Argentina	3
Brazil	Bioinformatics Laboratory—LNCC	Goiais	1
		Minas Gerais	5
		Rio de Janeiro	7
		Rio Grande do Sul	1
		São Paulo	4
	Instituto Adolfo Lutz, Interdiciplinary Procedures Center, Strategic Laboratory	Brasilia	1
		Sao Paulo	2
		Sao Paulo	11
	Instituto Oswaldo Cruz FIOCRUZ - Laboratory of Respiratory Viruses and Measles (LVRS)	Maceio	1
		Feira de Santana	2
		Brasilia	5
		Vila Velha	1
		Niteroi	1
		Rio de Janeiro	6
		Florianopolis	1
		Joinville	1
	Laboratorio de Ecologia de Doencas Transmissiveis na Amazonia, Instituto Leonidas e Maria Deane—Fiocruz Amazonia	Manaus	1
	Laboratory of Virology	Brasilia	1
Chile	Instituto de Salud Publica de Chile	Santiago	2
		Talca	2
	MSHS Pathogen Surveillance Program	Santiago	3
Colombia	Instituto Nacional de Salud Universidad Cooperativa de Colombia Instituto Alexander von Humboldt Imperial College-London London School of Hygiene & Tropical Medicine	Antioquia	1
		Bogota	1
Ecuador	Institute of Microbiology, Universidad San Francisco de Quito	Pichincha	3
		Quito	1
Mexico	Instituto de Diagnostico y Referencia Epidemiologicos (INDRE)	Chiapas	1
		Estado de Mexico	1
		Mexico City	2
		Puebla	1
		Queretaro	1
	Instituto Nacional de Ciencias Medicas y Nutricion Salvador Zubiran	Mexico City	5
	Instituto Nacional de Enfermedades Respiratorias	Mexico City	5
	Laboratorio Central de Epidemiología-DLVIE / Laboratorio de Secuenciación-Centro de Instrumentos. Instituto Mexicano del Seguro Social	Chihuahua	1
Nepal^a^	The University of Hong Kong	Kathmandu	1
Panama	Gorgas Memorial Institute for Health Studies	Panama City	1
Peru	Laboratorio de Referencia Nacional de Biotecnologia y Biologia Molecular. Instituto Nacional de Salud Peru	Lima	2
Uruguay	Microbial Genomics Laboratory, Institut Pasteur Montevideo	Montevideo	9
Total general			98

**Note:**

a Nepal was included due to researchers with Colombian and Honduran affiliations contributed in its genome sequencing (Sah et al., 2020).

## Medical Contributions

### Epidemiological analyses

Studies to track first cases in different countries have been performed, such as the case of tracing the first cases of COVID-19 in countries like Italy (Giovanetti et al., 2020b) and Chile (Castillo et al., 2020) using phylogenetic analyses. Both studies found that the first cases came from China and Europe since S and G variants of SARS-CoV-2 were detected. In the case of Italy, a reentry from Germany was detected (Giovanetti et al., 2020a). Tracing of imported cases to other Latin American countries has also been performed using strategies such as data recovery of international flights from the most affected countries to Brazil (Candido et al., 2020), or recompilation of epidemiological data from hospitals in Bolivia (Escalera-Antezana et al., 2020). Both studies concluded that the first imported cases came from Europe, specifically from Italy and Spain. Moreover, other bioinformatics tools have been used to contribute to epidemiological understanding. For example, a Bayesian phylogeographic reconstruction suggested that Wuhan was effectively the outbreak epicenter on 25 November 2019, spreading later to other Chinese regions (Benvenuto et al., 2020c).

Additionally, the geo-positioning of some cases and the heterogeneity of the outbreak progress among countries, with demographic similarities, have been reported (De Figueiredo et al., 2020; Xu et al., 2020). Other epidemiological studies have been done to predict the COVID-19 spreading (Benvenuto et al., 2020d; Córdova-Lepe, Gutiérrez-Aguilar & Gutiérrez-Jara, 2020; González-Jaramillo et al., 2020; Manrique-Abril et al., 2020), which concluded that containment strategies are required to avoid overspreading in countries like Chile and Colombia. Similarly, Kraemer et al. (2020) analyzed the effect of mobility in China before and after the sanitary containment of Wuhan in the COVID-19 spreading in this country; they demonstrated that decreasing mobility favors the reduction of COVID-19 spreading.

### Meta-analyses performed

Latin American researchers have also published meta-analyses of clinical, laboratory and image data from reported COVID-19 cases (Do Nascimento et al., 2020; Rodriguez-Morales et al., 2020b). On the one hand, the meta-analysis submitted by Rodriguez-Morales et al. (2020b) on 29 February 2020, used 19 articles with 2,874 patients for quantitative analyses. On the other hand, the meta-analysis of Do Nascimento et al. (2020), submitted 6 days later, employed 61 studies with 59,254 patients. Both revisions included the most common symptoms as well as typical abnormalities observed in chest radiographs and computed tomographies, which were like other respiratory illnesses and viral pneumonia, making it difficult to distinguish from them (Do Nascimento et al., 2020). Oxygen support in critical patients was also analyzed, where Do Nascimento et al. (2020) suggested that excluding the non-invasive ventilation usage since no evidence supports its benefits.

Regarding lethality, the most affected population (81%) was the older group (60 years or more), but additional information is required to understand the COVID-19 impact on other continents because most of the data used in these meta-analyses came from China. Therefore, meta-analyses using Latin American cases would also be ideal for determining how COVID-19 could affect this region, which has some differences, such as lower average age or higher exposure to respiratory infections than other regions like Europe (Amariles et al., 2020a). A third meta-analysis was found, which suggested that it could be possible to predict if a patient with COVID-19 can present complications. Lagunas-Rangel (2020) suggested that complications are related to high levels of neutrophil-to-lymphocyte ratio (NLR) and low levels of lymphocyte-to-C-reactive protein ratio (LCR).

### Search for potential treatments

Concerning treatments to respiratory issues, Khoury et al. (2020) revised respiratory therapies for COVID-19 patients using cell-based treatments such as mesenchymal stem cells (MSCs), derivatives, or other cells, which have shown positive results in pre-clinical models of influenza. Conversely, they argued that there were few coronavirus studies, such as the case of seven COVID-19 patients in Beijing treated with MSCs, who showed apparent improvements up to 4 days after treatment, but lacked detailed information (Leng et al., 2020). Therefore, after a systematic search in databases, Khoury et al. (2020) found 27 ongoing treatments with 1,287 patients. The authors also highlighted the importance of following ethical protocols for these types of treatments.

Besides the previously mentioned review, since currently there are no approved treatments or vaccines for COVID-19, other revisions have summarized the development of clinical trials for COVID-19 treatments. This is the case of the search made by Rosa & Santos (2020), who looked for ongoing clinical trials in Clinicaltrials.gov. The authors used some constraints in their search, such as low cost, reduced time to reach markets, existing pharmaceutical supplies, or the possibility to combine with other drugs. In total, 24 clinical trials were found, most of them in clinical phases 2, 3, or 4, with a scheduled end in 2020. These ongoing clinical trials are using chloroquine, hydroxychloroquine, human immunoglobulin, remdesivir, arbidol, lopinavir, ritonavir, oseltamivir, darunavir, cobicistat, interferons, carrimycin, danoprevir, xiyanping, favipiravir, thalidomide, vitamin C, methylprednisolone, pirfenidone, bromhexie, bevacizumab, fingolimod, and traditional Chinese medicines (TCM).

Similarly, Serafin et al. (2020) searched for new treatments for coronaviruses like SARS-CoV, SARS-CoV-2 and HCoV-OC43 in PubMed, SCOPUS, and Web of Science databases. The drugs found as SARS-CoV-2 treatments are captopril, chloroquine, clomipramine, disulfiram, enalapril, hydroxychloroquine, mefloquine, metformin, nitazoxanide, remdesivir and teicoplanin. This review highlighted positive results using chloroquine, hydroxychloroquine, teicoplanin and hydroxychloroquine in conjunction with azithromycin. Finally, Rosales-Mendoza (2020) suggested that a potential treatment with biotechnological origin could be produced using plant species as hosts.

### Medical considerations

Most medical articles studied populations at risk and comorbidities, also provided recommendations and guidelines for medical personnel. Rascado Sedes et al. (2020) developed a contingency plan that allows an optimal response of the intensive care units (ICU) to the pandemic. The plan considers possible scenarios, the need for human and technical resources, communication and information, optimized use of resources, and personal protective equipment (PPE).

Healthcare workers, emergency room physicians, anesthesiologists, dentists, ophthalmologists, head and neck surgeons, maxillofacial surgeons, and otolaryngologists are among the most vulnerable ones because they perform procedures that can aerosolize secretions (Kowalski et al., 2020). According to several studies, articles, and protocols, Boccalatte et al. (2020) summarized recommendations related to PPE, mandatory use of protective suits, head covers, eye protection, mask, gloves, and N95, FFP2, or PAPR masks, personnel training, and techniques or manoeuvers for different medical practices.

Concerning surgeries, although there is no information about to translate risks to the operating room team during such procedures to COVID-19 patients, the recommendation is to postpone them (Cohen et al., 2020; Ducournau et al., 2020). The situations in which a delay in the surgical procedure may affect the patient, such as some oncologic or organ transplant surgeries, surgery must be performed following strict preventive measures. In the case of unpostponable abdominal surgeries, a laparotomic operation with regional anesthesia should be preferred (Carneiro et al., 2020; Cohen et al., 2020; Correia, Ramos & Bahten, 2020; Quintão et al., 2020). Other articles summarized protocols and recommendations for hand (Ducournau et al., 2020), head and neck (Kowalski et al., 2020), and urologic surgeries (Puliatti et al., 2020).

Researchers have also performed literature reviews concerning populations with higher risk due to comorbidities. First, Hussain, Bhowmik & Do Vale Moreira (2020) focused on patients with diabetes mellitus and COVID-19. They summarized the different determinants that associate this pathology with greater severity and death, as well as the importance of multidisciplinary medical management. The authors mentioned that COVID-19 could cause pancreatic damage, which could affect patients with diabetes. Likewise, the positive effects of hydroxychloroquine and chloroquine on diabetic patients were mentioned, such as reducing insulin degradation tending to normalize glucose levels. Therefore, in case hydroxychloroquine or chloroquine is administered, antidiabetic drug doses should be readjusted to avoid hypoglycemic events. The authors also mentioned the necessity to continue studying in these patients the chronic inflammation, immune response, coagulation activity and vascular permeability, as well as whether hyperglycemia or hypoglycemia can alter the virulence of SARS-CoV-2, or whether the virus itself interferes with insulin secretion or glycemic control. All this information will be needed to propose adequate clinical treatments.

Likewise, Puliatti et al. (2020) considered the effect of SARS-CoV-2 in different organs of the urinary tract. They highlighted that ACE2-positive cells (target of SARS-CoV-2 spike proteins) have been found in these organs, which could have a high risk of affectation, even leading to death, which explains the kidney damage experimented in some COVID-19 patients. Similarly, chronic hemodialysis patients are also at particular risk due to their immunosuppression status, advanced age, and comorbidities coexistence. Vega-Vega et al. (2020) summarized the recommendations for these patients proposed by three international organizations: Center for Disease Control and Prevention (CDC), the Spanish Society of Nephrology, and the Latin American Society of Nephrology and Hypertension, to which were added suggestions of some experts. Some recommendations are the proper definition of cases, guidelines for patients and family, scrutiny of suspicious COVID-19 cases and their management within hemodialysis units, PPE employment, and sanitation of surfaces and devices.

Another group at risk mentioned by researchers is related to critical COVID-19 patients who need assisted ventilation. Some authors recommend taking into account some considerations before using high-flow nasal oxygen therapy, non-invasive ventilation, or extracorporeal membrane oxygenation, the latter being a treatment that no all medical centers can afford. First, if the medical center has adequate protection levels for health workers from exhaled air dispersed, and second, the impact of treatment on acute respiratory distress syndrome (Bartlett et al., 2020; Ñamendys-Silva, 2020a, 2020b). Similarly, Chica-Meza et al. (2020) summarized conventional and non-conventional respiratory therapies in critical COVID-19 patients, including recommendations and considerations. Finally, surveys have been performed to identify COVID-19 impacts in patients at risk, such as pediatric patients with cancer (Hrusak et al., 2020), but the information is still limited; however, the recommendation is to follow the same medical practices described previously.

### Mental health considerations

Mental health care must be considered, given that quarantine can cause boredom, loneliness, anger, anxiety, depression and stress. Patients and health workers should have mental health services (De Medeiros Carvalho et al., 2020b; Lima et al., 2020). In especial when more people can be affected by mental issues during the outbreak than by the outbreak itself (Ornell et al., 2020), and previous outbreaks have caused post-traumatic stress disorder in health workers (Torales et al., 2020).

Concerning performed studies on mental issues, first, Fonseca et al. (2020) established recommendations for patients with schizophrenia. Among recommendations are an adequate identification of COVID-19 symptoms, prevention of worsening of psychiatric symptoms, relapses due to the closed environment, fear of disease and isolation, use of telemedicine, promoting adherence to antipsychotic medication regimens, reducing emotional distress, hygiene practices, and family support. Finally, Carvalho, Pianowski & Gonçalves (2020a), using questionnaires to Brazilians, investigated whether extroverted and conscientious people are engaged with the containment measures implemented during the COVID-19 pandemic. They found that extroverted people seem to lack commitment to containment measures, while people with conscientiousness personality tend to follow recommendations. Therefore, at least in Brazil, strategies for extroverted people should be proposed to avoid they become transmission vectors. However, the idiosyncrasy is similar throughout Latin America; thus, these strategies should be required in the entire region.

## Additional Concerns

### Social and environmental concerns

- Telemedicine should be considered as an alternative to patient attention to avoid COVID-19 spreading. However, this strategy is limited because of the scarce existence of telemedicine systems; thus, social media could be used (Machado et al., 2020; Quintão et al., 2020).
- Elachola, Ebrahim & Gozzer (2020) highlighted that there are no prevalence and effectiveness studies on using facemasks related to the COVID-19 outbreak. This information could be useful in providing recommendations about their usage. However, based on past outbreaks, the evidence did not show positive or negative effects when the population used facemasks (Stern et al., 2020).
- Since the first COVID-19 cases reported in Latin America, fake news and misinformation have increased, where even treatments with potential health affectations have been proposed, as in the case of using chloroform or ether as alleged COVID-19 treatments (Lana et al., 2020; Martins & Santos, 2020). Additionally, information should be verified, avoiding panic spreading, which has caused panic buying of supplies and medications (Cuan-Baltazar et al., 2020).
- The quick COVID-19 spreading in different regions and countries could cause that health workers to become overwhelmed, therefore training new personal, although challenging, is a necessity that could help to attend the current outbreak. Additionally, the pharmacy workforce should be prepared as they are the first ones to attend possible cases of infection (Amariles et al., 2020b; Aruru, Truong & Clark, 2020; Haines et al., 2020).
- Countries with low or middle incomes, such as Latin American countries, may have already saturated their health systems. Hence, the COVID-19 outbreak could oversaturate them (Carneiro et al., 2020), which can be exacerbated by the lack of government preparation and social policies, such as the Ecuadorian case (Hallo, Rojas & Hallo, 2020).
- Based on events that occurred with the “Diamond Princess” cruise, alternatives should be proposed to quarantine people from other cruises and illegal immigrants entering a country (Sawano et al., 2020).
- Due to the current outbreak, many activities have been interrupted, which joined the socioeconomic limitations of vulnerable groups, could lead to food insecurity in these populations, who even have no access to clean water (Oliveira, Abranches & Lana, 2020).
- Knowing that COVID-19 has a zoonotic origin, One Health approach has taken relevance, which seeks integrative studies where the health of humans, environment and animals are considered to understand the virus environment, allowing the prevention or mitigation of future outbreaks (Bonilla-Aldana, Dhama & Rodriguez-Morales, 2020a).

### Medical concerns

- Initial reports of some pathologies such as rheumatic diseases or neuromyelitis optica spectrum disorder (NMOSD) have shown that they do not increase the risk of complications like other comorbidities. However, further studies are needed (Carnero Contentti & Correa, 2020; Figueroa-Parra et al., 2020).
- Angiotensin II receptor blockers (ATII-RB) are hypertensive drugs that increase ACE2 expression; therefore, there is a possibility that they can favor the internalization of SARS-CoV-2 within the cell; thus, further studies are required. However, suspending ATII-RB therapy may cause even higher affectations than COVID-19 itself; the risk-benefit ratio should be evaluated. In case doctors consider suspending it, there are other options like thiazide diuretics drugs (Gracia-Ramos, 2020; Marin, 2020).
- As previously mentioned, chloroquine and hydroxychloroquine are in vitro inhibitors of SARS-CoV-2 infection. However, concerns are focused on whether these medications could decrease viral load or prevent infection, clinical disease, clinical severity, or even death. Other factors, such as side effects, should also be considered (Kim et al., 2020; Monteiro et al., 2020; Picot et al., 2020).
- COVID-19 mainly affects the respiratory system, but it can also alter the central nervous system. Hence, possible affectations in the respiratory center could exacerbate respiratory distress caused by pulmonary affectation (Conde et al., 2020).
- Tropical countries affected by dengue could face two outbreaks at the same time, dengue and COVID-19, which could affect the population, even coinfecting some patients simultaneously. Both outbreaks will require the intensive attention of health systems to avoid a crossed affectation between them, which can be challenging and overwhelming for the health systems (Lorenz, Azevedo & Chiaravalloti-Neto, 2020; Navarro et al., 2020; Rodriguez-Morales, Sah & Paniz-Mondolfi, 2020; Saavedra-Velasco et al., 2020).
- As SARS-CoV-2 can remain in saliva, oral health professionals require research focused on the influence of COVID-19 in their activities to take appropriate measures. However, the recommendation is to stop dental treatments (Martelli-Júnior et al., 2020; Napimoga & Freitas, 2020; Sabino-Silva, Jardim & Siqueira, 2020).
- Strategies during intubation are not the only important ones to avoid COVID-19 spreading in health workers during medical procedures; strategies for extubation are also required (Trujillo, 2020).
- There is no evidence that immunosuppressant treatments could decrease or increase the risk of severe COVID-19 infection; therefore, further investigation is recommended. In case treatments are suspended, factors such as potential issues on patients should be considered (Carnero Contentti & Correa, 2020).
- There is limited evidence of COVID-19 effect on pregnant women; hence, cases of pregnant women with COVID-19 should be studied to understand the clinical impact of the infection (Zambrano et al., 2020).

## Conclusions

Although our purpose was to give visibility to the contribution of Latin American researchers in the knowledge generation related to the COVID-19 outbreak, this review has two drawbacks. The first is the continuous availability of new publications; therefore, an observation window was employed. Second, several Latin American researchers are currently working on other continents without a Latin American affiliation, making them impossible to track. However, after this literature review, we were able to evidence the active participation of Latin American researchers in different subjects, whether as members of national, regional (LANCOVID-19), or even international research groups. Concerning our findings, the publications evidenced that these research groups have advanced in molecular and medical subjects, mainly in genetic understanding, epidemiological behaviors, meta-analyses, interaction between COVID-19 and other pathologies, and recommendations to medical procedures. Finally, understanding that this health crisis requires the commitment of as many researchers as possible, our wish is that the contribution of Latin American researchers continues to grow. Some topics with regional and global interest for future studies include in silico analyses of potential treatments and their respective in vitro and in vivo validations, meta-analysis of Latin American patients, and epidemiological surveillances. Regarding medical considerations, a deeper understanding of the COVID-19 interaction with risk comorbidities is needed to propose adequate clinical treatments. The same applies to unexplored/underexplored physical and mental pathologies, such as dengue.

## Supplemental Information

10.7717/peerj.9332/supp-1 Supplemental Information 1 PRISMA checklist.

10.7717/peerj.9332/supp-2 Supplemental Information 2 COVID-19 or SARS-CoV-2 publications with at least one researcher with Latin American affiliation.

10.7717/peerj.9332/supp-3 Supplemental Information 3 Genome sequences from Latin American SARS-CoV-2 strains collected in the GISAID database.

## Additional Information and Declarations

### Data Availability

The following information was supplied regarding data availability: Raw data are available in the Supplemental Files.

## References

[ref-1] Amariles P, Granados J, Ceballos M, Montoya CJ (2020a). COVID-19 in Colombia endpoints: are we different, like Europe?. Research in Social and Administrative Pharmacy.

[ref-2] Amariles P, Ledezma-Morales M, Salazar-Ospina A, Hincapié-García JA (2020b). How to link patients with suspicious COVID-19 to health system from the community pharmacies? A route proposal. Research in Social and Administrative Pharmacy.

[ref-3] Andersen KG, Rambaut A, Lipkin WI, Holmes EC, Garry RF (2020). The proximal origin of SARS-CoV-2. Nature Medicine.

[ref-4] Angeletti S, Benvenuto D, Bianchi M, Giovanetti M, Pascarella S, Ciccozzi M (2020). COVID-2019: the role of the nsp2 and nsp3 in its pathogenesis. Journal of Medical Virology.

[ref-5] Aruru M, Truong H-A, Clark S (2020). Pharmacy emergency preparedness and response (PEPR) framework for expanding pharmacy professionals’ roles and contributions to emergency preparedness and response during the COVID-19 pandemic and beyond. Research in Social and Administrative Pharmacy.

[ref-6] Bartlett RH, Ogino MT, Brodie D, McMullan DM, Lorusso R, MacLaren G, Stead CM, Rycus P, Fraser JF, Belohlavek J, Salazar L, Mehta Y, Raman L, Paden ML (2020). Initial ELSO guidance document: ECMO for COVID-19 patients with severe cardiopulmonary failure. ASAIO Journal: Artificial Organ Research and Development.

[ref-7] Benvenuto D, Angeletti S, Giovanetti M, Bianchi M, Pascarella S, Cauda R, Ciccozzi M, Cassone A (2020a). Evolutionary analysis of SARS-CoV-2: how mutation of non-structural protein 6 (NSP6) could affect viral autophagy. Journal of Infection.

[ref-8] Benvenuto D, Giovanetti M, Ciccozzi A, Spoto S, Angeletti S, Ciccozzi M (2020b). The 2019-new coronavirus epidemic: evidence for virus evolution. Journal of Medical Virology.

[ref-9] Benvenuto D, Giovanetti M, Salemi M, Prosperi M, De Flora C, Alcantara LC, Angeletti S, Ciccozzi M (2020c). The global spread of 2019-nCoV: a molecular evolutionary analysis. Pathogens and Global Health.

[ref-10] Benvenuto D, Giovanetti M, Vassallo L, Angeletti S, Ciccozzi M (2020d). Application of the ARIMA model on the COVID-2019 epidemic dataset. Data in Brief.

[ref-11] Boccalatte LA, Larrañaga JJ, Perez Raffo GM, Teijido CA, García Fornari G, Staneloni MI, Figari MF (2020). Brief guideline for the prevention of COVID-19 infection in head and neck and otolaryngology surgeons. American Journal of Otolaryngology.

[ref-12] Bonilla-Aldana DK, Dhama K, Rodriguez-Morales AJ (2020a). Revisiting the one health approach in the context of COVID-19: a look into the ecology of this emerging disease. Advances in Animal and Veterinary Sciences.

[ref-13] Bonilla-Aldana DK, Quintero-Rada K, Montoya-Posada JP, Ramirez-Ocampo S, Paniz-Mondolfi A, Rabaan AA, Sah R, Rodriguez-Morales AJ (2020b). SARS-CoV, MERS-CoV and now the 2019-novel CoV: have we investigated enough about coronaviruses?—A bibliometric analysis. Travel Medicine and Infectious Disease.

[ref-14] Candido DDS, Watts A, Abade L, Kraemer MUG, Pybus OG, Croda J, De Oliveira W, Khan K, Sabino EC, Faria NR (2020). Routes for COVID-19 importation in Brazil. Journal of Travel Medicine.

[ref-15] Carneiro A, Wroclawski M, Nahar B, Soares A, Cardoso A, Kim N, Carvalho F (2020). Impact of the COVID-19 Pandemic on the Urologist’s clinical practice in Brazil: a management guideline proposal for low- and middle-income countries during the crisis period. International Brazilian Journal of Urology.

[ref-16] Carnero Contentti E, Correa J (2020). Immunosuppression during the COVID-19 pandemic in neuromyelitis optica spectrum disorders patients: a new challenge. Multiple Sclerosis and Related Disorders.

[ref-17] Carvalho LdF, Pianowski G, Gonçalves AP (2020a). Personality differences and COVID-19: are extroversion and conscientiousness personality traits associated with engagement with containment measures?. Trends in Psychiatry and Psychotherapy.

[ref-18] Castillo AE, Parra B, Tapia P, Acevedo A, Lagos J, Andrade W, Arata L, Leal G, Barra G, Tambley C, Tognarelli J, Bustos P, Ulloa S, Fasce R, Fernández J (2020). Phylogenetic analysis of the first four SARS-CoV-2 cases in Chile. Journal of Medical Virology.

[ref-19] Chica-Meza C, Peña-López LA, Villamarín-Guerrero HF, Moreno-Collazos JE, Rodríguez-Corredor LC, Lozano WM, Vargas-Ordoñez MP (2020). Cuidado respiratorio en Covid-19. Acta Colombiana de Cuidado Intensivo.

[ref-20] Ciccozzi M, Benvenuto D, Giovanetti M, Bianchi M, Pascarella S, Angeletti S (2020). Response to Ribeiro da Silva etal,“Role of Nonstructural Proteins in the Pathogenesis of SARS-CoV-2. Journal of Medical Virology.

[ref-21] Ciotti M, Angeletti S, Minieri M, Giovannetti M, Benvenuto D, Pascarella S, Sagnelli C, Bianchi M, Bernardini S, Ciccozzi M (2020). COVID-19 outbreak: an overview. Chemotherapy.

[ref-22] Cleemput S, Dumon W, Fonseca V, Karim WA, Giovanetti M, Alcantara LC, Deforche K, De Oliveira T (2020). Genome detective coronavirus typing tool for rapid identification and characterization of novel coronavirus genomes. Bioinformatics.

[ref-23] Cohen SL, Liu G, Abrao M, Smart N, Heniford T (2020). Perspectives on surgery in the time of COVID-19: safety first. Journal of Minimally Invasive Gynecology.

[ref-24] Conde G, Quintana Pájaro LD, Quintero Marzola ID, Villegas YR, Moscote Salazar LR (2020). Neurotropism of SARS-CoV 2: mechanisms and manifestations. Journal of the Neurological Sciences.

[ref-25] Correia MITD, Ramos RF, Bahten LCV (2020). Os cirurgiões e a pandemia do COVID-19. Revista do Colégio Brasileiro de Cirurgiões.

[ref-26] Cuan-Baltazar JY, Muñoz-Perez MJ, Robledo-Vega C, Pérez-Zepeda MF, Soto-Vega E (2020). Misinformation of COVID-19 on the internet: infodemiology study. JMIR Public Health Surveill.

[ref-27] Cupertino MC, Resende MB, Mayer NAJ, Carvalho LM, Siqueira-Batista R (2020). Emerging and re-emerging human infectious diseases: a systematic review of the role of wild animals with a focus on public health impact. Asian Pacific Journal of Tropical Medicine.

[ref-28] Cárdenas‐Conejo Y, Liñan‐Rico A, García‐Rodríguez DA, Centeno‐Leija S, Serrano‐Posada H (2020). An exclusive 42 amino acid signature in pp1ab protein provides insights into the evolutive history of the 2019 novel human‐pathogenic coronavirus (SARS‐CoV‐2). Journal of Medical Virology.

[ref-29] Córdova-Lepe F, Gutiérrez-Aguilar R, Gutiérrez-Jara JP (2020). Number of COVID-19 cases in Chile at 120 days with data at 21/03/2020 and threshold of daily effort to flatten the epi-curve. Medwave.

[ref-30] De Figueiredo AM, Codina AD, De Figueiredo DCMM, Gil-García E, Kalache A (2020). Letalidad del COVID-19: ausencia de patrón epidemiológico. Gaceta Sanitaria.

[ref-31] De Medeiros Carvalho PM, Moreira MM, De Oliveira MNA, Landim JMM, Neto MLR (2020b). The psychiatric impact of the novel coronavirus outbreak. Psychiatry Research.

[ref-32] Diaz-Quijano FA, Rodriguez-Morales AJ, Waldman EA (2020). Translating transmissibility measures into recommendations for coronavirus prevention. Revista de Saúde Pública.

[ref-33] Do Nascimento IJB, Cacic N, Abdulazeem MH, Von Groote CT, Jayarajah U, Weerasekara I, Esfahani AM, Civile TV, Marusic A, Jeroncic A, Carvas Junior N, Pericic PT, Zakarija-Grkovic I, Meirelles Guimarães MS, Luigi Bragazzi N, Bjorklund M, Sofi-Mahmudi A, Altujjar M, Tian M, Arcani MD, O’Mathúna PD, Marcolino SM (2020). Novel coronavirus infection (COVID-19) in humans: a scoping review and meta-analysis. Journal of Clinical Medicine.

[ref-34] Ducournau F, Arianni M, Awwad S, Baur EM, Beaulieu JY, Bouloudhnine M, Caloia M, Chagar K, Chen Z, Chin AY, Chow EC, Cobb T, David Y, Delgado PJ, Woon Man Fok M, French R, Golubev I, Haugstvedt JR, Ichihara E, Jorquera RA, Koo SCJJ, Lee JY, Lee YK, Lee YJ, Liu B, Kaleli T, Mantovani GR, Mathoulin C, Messina JC, Muccioli C, Nazerani S, Ng CY, Obdeijn MC, Van Overstraeten L, Prasetyono TOH, Ross M, Shih JT, Smith N, Suarez RFA, Chan PT, Tiemdjo H, Wahegaonkar A, Wells MC, Wong WY, Wu F, Yang XF, Yanni D, Yao J, Liverneaux PA (2020). COVID-19: initial experience of an international group of hand surgeons. Hand Surgery and Rehabilitation.

[ref-35] Elachola H, Ebrahim SH, Gozzer E (2020). COVID-19: facemask use prevalence in international airports in Asia, Europe and the Americas, March 2020. Travel Medicine and Infectious Disease.

[ref-36] Escalera-Antezana JP, Lizon-Ferrufino NF, Maldonado-Alanoca A, De-la-Vega GA, Alvarado-Arnez LE, Balderrama-Saavedra MA, Bonilla-Aldana DK, Rodríguez-Morales AJ (2020). Clinical features of the first cases and a cluster of coronavirus disease 2019 (COVID-19) in Bolivia imported from Italy and Spain. Travel Medicine and Infectious Disease.

[ref-37] Figueroa-Parra G, Aguirre-Garcia GM, Gamboa-Alonso CM, Camacho-Ortiz A, Galarza-Delgado DA (2020). Are my patients with rheumatic diseases at higher risk of COVID-19?. Annals of the Rheumatic Diseases.

[ref-38] Fonseca L, Diniz E, Mendonça G, Malinowski F, Mari J, Gadelha A (2020). Schizophrenia and COVID-19: risks and recommendations. Brazilian Journal of Psychiatry.

[ref-39] Giovanetti M, Angeletti S, Benvenuto D, Ciccozzi M (2020a). A doubt of multiple introduction of SARS-CoV-2 in Italy: a preliminary overview. Journal of Medical Virology.

[ref-40] Giovanetti M, Benvenuto D, Angeletti S, Ciccozzi M (2020b). The first two cases of 2019-nCoV in Italy: where they come from?. Journal of Medical Virology.

[ref-41] González-Jaramillo V, González-Jaramillo N, Gómez-Restrepo C, Palacio-Acosta CA, Gómez-López A, Franco OH (2020). Proyecciones de impacto de la pandemia COVID-19 en la población colombiana, según medidas de mitigación. Datos preliminares de modelos epidemiológicos para el periodo del 18 de marzo al 18 de abril de 2020. Revista de Salud Pública.

[ref-42] Gracia-Ramos AE (2020). Is the ACE2 Overexpression a Risk Factor for COVID-19 Infection?. Archives of Medical Research.

[ref-43] Hadfield J, Megill C, Bell SM, Huddleston J, Potter B, Callender C, Sagulenko P, Bedford T, Neher RA (2018). NextStrain: real-time tracking of pathogen evolution. Bioinformatics.

[ref-44] Haines A, De Barros EF, Berlin A, Heymann DL, Harris MJ (2020). National UK programme of community health workers for COVID-19 response. Lancet.

[ref-45] Hallo A, Rojas A, Hallo C (2020). Perspective from ecuador, the second country with more confirmed cases of coronavirus disease 2019 in south america: a review. Cureus.

[ref-46] Hrusak O, Kalina T, Wolf J, Balduzzi A, Provenzi M, Rizzari C, Rives S, Del Pozo Carlavilla M, Valerio Alonso ME, Domínguez Pinilla N, Bourquin J-P, Schmiegelow K, Attarbaschi A, Grillner P, Mellgren K, Ten Bosch Van Der Werff J, Pieters R, Brozou T, Borkhardt A, Escherich G, Lauten M, Stanulla M, Smith O, Juh Yeoh AE, Elitzur S, Vora A, Li C-K, Ariffin H, Kolenova A, Dallapozza L, Farah R, Lazic J, Manabe A, Styczynski J, Kovacs G, Ottoffy G, Felice M, Buldini B, Conter V, Stary J, Schrappe M (2020). Flash survey on SARS-CoV-2 infections in pediatric patients on anti-cancer treatment. European Journal of Cancer.

[ref-47] Huang C, Wang Y, Li X, Ren L, Zhao J, Hu Y, Zhang L, Fan G, Xu J, Gu X, Cheng Z, Yu T, Xia J, Wei Y, Wu W, Xie X, Yin W, Li H, Liu M, Xiao Y, Gao H, Guo L, Xie J, Wang G, Jiang R, Gao Z, Jin Q, Wang J, Cao B (2020). Clinical features of patients infected with 2019 novel coronavirus in Wuhan, China. Lancet.

[ref-48] Hussain A, Bhowmik B, Do Vale Moreira NC (2020). COVID-19 and diabetes: knowledge in progress. Diabetes Research and Clinical Practice.

[ref-49] Johns Hopkins University (2020). COVID-19 dashboard by the center for systems science and engineering (CSSE). https://coronavirus.jhu.edu/map.html.

[ref-50] Khoury M, Cuenca J, Cruz FF, Figueroa FE, Rocco PRM, Weiss DJ (2020). Current status of cell-based therapies for respiratory virus infections: applicability to COVID-19. European Respiratory Journal.

[ref-51] Kim AHJ, Sparks JA, Liew JW, Putman MS, Berenbaum F, Duarte-García A, Graef ER, Korsten P, Sattui SE, Sirotich E, Ugarte-Gil MF, Webb K, Grainger R, Alliance† C-GR (2020). A rush to judgment? Rapid reporting and dissemination of results and its consequences regarding the use of hydroxychloroquine for COVID-19. Annals of Internal Medicine.

[ref-52] Kowalski LP, Sanabria A, Ridge JA, Ng WT, De Bree R, Rinaldo A, Takes RP, Mäkitie AA, Carvalho AL, Bradford CR, Paleri V, Hartl DM, Poorten VV, Nixon IJ, Piazza C, Lacy P, Rodrigo JP, Guntinas-Lichius O, Mendenhall WM, D’Cruz A, Lee AWM, Ferlito A (2020). COVID-19 pandemic: effects and evidence-based recommendations for otolaryngology and head and neck surgery practice. Head & Neck.

[ref-53] Kraemer MUG, Yang C-H, Gutierrez B, Wu C-H, Klein B, Pigott DM, Du Plessis L, Faria NR, Li R, Hanage WP, Brownstein JS, Layan M, Vespignani A, Tian H, Dye C, Pybus OG, Scarpino SV (2020). The effect of human mobility and control measures on the COVID-19 epidemic in China. Science.

[ref-54] Lagunas-Rangel FA (2020). Neutrophil-to-Lymphocyte ratio and Lymphocyte-to-C-reactive protein ratio in patients with severe coronavirus disease 2019 (COVID-19): a meta-analysis. Journal of Medical Virology.

[ref-55] Lana RM, Coelho FC, Gomes MFDC, Cruz OG, Bastos LS, Villela DAM, Codeço CT (2020). The novel coronavirus (SARS-CoV-2) emergency and the role of timely and effective national health surveillance. Cadernos de saude publica.

[ref-56] Leng Z, Zhu R, Hou W, Feng Y, Yang Y, Han Q, Shan G, Meng F, Du D, Wang S, Fan J, Wang W, Deng L, Shi H, Li H, Hu Z, Zhang F, Gao J, Liu H, Li X, Zhao Y, Yin K, He X, Gao Z, Wang Y, Yang B, Jin R, Stambler I, Lim LW, Su H, Moskalev A, Cano A, Chakrabarti S, Min K-J, Ellison-Hughes G, Caruso C, Jin K, Zhao RC (2020). Transplantation of ACE2(−) mesenchymal stem cells improves the outcome of patients with COVID-19 pneumonia. Aging and Disease.

[ref-57] Lima CKT, Carvalho PMDM, Lima IDAAS, Nunes JVADO, Saraiva JS, De Souza RI, Da Silva CGL, Neto MLR (2020). The emotional impact of Coronavirus 2019-nCoV (new Coronavirus disease). Psychiatry Research.

[ref-58] Lorenz C, Azevedo TS, Chiaravalloti-Neto F (2020). COVID-19 and dengue fever: a dangerous combination for the health system in Brazil. Travel Medicine and Infectious Disease.

[ref-59] Machado RA, De Souza NL, Oliveira RM, Martelli Júnior H, Bonan PRF (2020). Social media and telemedicine for oral diagnosis and counselling in the COVID-19 Era. Oral Oncology.

[ref-60] Manrique-Abril FG, Agudelo-Calderon CA, González-Chordá VM, Gutiérrez-Lesmes O, Téllez-Piñerez CF, Herrera-Amaya G (2020). Modelo SIR de la pandemia de COVID-19 en Colombia. Revista de Salud Pública.

[ref-61] Marin GH (2020). Facts and reflections on COVID-19 and anti-hypertensives drugs. Drug Discoveries & Therapeutics.

[ref-62] Martelli-Júnior H, Machado RA, Martelli DRB, Coletta RD (2020). Dental journals and coronavirus disease (COVID-19): a current view. Oral Oncology.

[ref-63] Martins PR, Santos VS (2020). No evidence supports the use of ether and chloroform inhalation for treating COVID-19. Revista Panamericana de Salud Publica-Pan American Journal of Public Health.

[ref-64] Millán-Oñate J, Rodriguez-Morales AJ, Camacho-Moreno G, Mendoza-Ramírez H, Rodríguez-Sabogal IA, Álvarez-Moreno C (2020). A new emerging zoonotic virus of concern: the 2019 novel coronavirus (COVID-19). Infectio.

[ref-65] Monteiro WM, Brito-Sousa JD, Da-Silva DB, De Melo GC, Siqueira AM, Val F, Daniel-Ribeiro CT, Guimarães Lacerda MV (2020). Driving forces for COVID-19 clinical trials using chloroquine: the need to choose the right research questions and outcomes. Revista da Sociedade Brasileira de Medicina Tropical.

[ref-101] Ñamendys-Silva SA (2020a). ECMO for ARDS due to COVID-19. Heart & Lung.

[ref-102] Ñamendys-Silva SA (2020b). Respiratory support for patients with COVID-19 infection. Lancet Respiratory Medicine.

[ref-66] Napimoga MH, Freitas ARR (2020). Dentistry vs severe acute respiratory syndrome Coronavirus 2: how to face this enemy. RGO: Revista Gaúcha de Odontologia.

[ref-67] Navarro J-C, Arrivillaga-Henríquez J, Salazar-Loor J, Rodriguez-Morales AJ (2020). COVID-19 and dengue, co-epidemics in Ecuador and other countries in Latin America: pushing strained health care systems over the edge. Travel Medicine and Infectious Disease.

[ref-68] Oliveira TC, Abranches MV, Lana RM (2020). (In)Segurança alimentar no contexto da pandemia por SARS-CoV-2. Cadernos de Saude Publica.

[ref-69] Ornell F, Schuch JB, Sordi AO, Kessler FHP (2020). Pandemic fear” and COVID-19: mental health burden and strategies. Brazilian Journal of Psychiatry.

[ref-70] Ortega JT, Serrano ML, Pujol FH, Rangel HR (2020a). Role of changes in SARS-CoV-2 spike protein in the interaction with the human ACE2 receptor: an in silico analysis. EXCLI Journal.

[ref-71] Ortega JT, Serrano ML, Pujol FH, Rangel HR (2020b). Unrevealing sequence and structural features of novel coronavirus using in silico approaches: the main protease as molecular target. EXCLI Journal.

[ref-72] Palacios Cruz M, Santos E, Velázquez Cervantes MA, León Juárez M (2020). COVID-19, una emergencia de salud pública mundial. Revista Clínica Española.

[ref-73] Picot S, Marty A, Bienvenu A-L, Blumberg LH, Dupouy-Camet J, Carnevale P, Kano S, Jones MK, Daniel-Ribeiro CT, Mas-Coma S (2020). Coalition: advocacy for prospective clinical trials to test the post-exposure potential of hydroxychloroquine against COVID-19. One Health.

[ref-74] Puliatti S, Eissa A, Eissa R, Amato M, Mazzone E, Dell’Oglio P, Sighinolfi MC, Zoeir A, Micali S, Bianchi G, Patel V, Wiklund P, Coelho RF, Bernhard J-C, Dasgupta P, Mottrie A, Rocco B (2020). COVID-19 and urology: a comprehensive review of the literature. BJU International.

[ref-75] Quintão V, Simões CM, Navarro LHC, De Barros GAM, Salgado-Filho MF, Guimarães GMN, Alves RL, Caetano AMM, Schmidt AP, Carmona MJC (2020). The anesthesiologist and COVID-19. Brazilian Journal of Anesthesiology.

[ref-76] Rascado Sedes P, Ballesteros Sanz MA, Bodí Saera MA, Carrasco Rodríguez-Rey LF, Castellanos Ortega Á, Catalán González M, De Haro López C, Díaz Santos E, Escriba Barcena A, Frade Mera MJ, Igeño Cano JC, Martín Delgado MC, Martínez Estalella G, Raimondi N, Roca I.Gas O, Rodríguez Oviedo A, Romero San Pío E, Trenado Álvarez J, Raurell M, Junta directiva de la SEMICYUC and Junta directiva de la SEEIUC (2020). Plan de contingencia para los servicios de medicina intensiva frente a la pandemia COVID-19. Enfermería Intensiva.

[ref-77] Rodriguez-Morales AJ, Bonilla-Aldana DK, Tiwari R, Sah R, Rabaan AA, Dhama K (2020a). COVID-19, an emerging coronavirus infection: current scenario and recent developments—an overview. Journal of Pure and Applied Microbiology.

[ref-78] Rodriguez-Morales AJ, Cardona-Ospina JA, Gutiérrez-Ocampo E, Villamizar-Peña R, Holguin-Rivera Y, Escalera-Antezana JP, Alvarado-Arnez LE, Bonilla-Aldana DK, Franco-Paredes C, Henao-Martinez AF, Paniz-Mondolfi A, Lagos-Grisales GJ, Ramírez-Vallejo E, Suárez JA, Zambrano LI, Villamil-Gómez WE, Balbin-Ramon GJ, Rabaan AA, Harapan H, Dhama K, Nishiura H, Kataoka H, Ahmad T, Sah R, Latin American Network of Coronavirus Disease (2020b). Clinical, laboratory and imaging features of COVID-19: A systematic review and meta-analysis. Travel Medicine and Infectious Disease.

[ref-79] Rodriguez-Morales AJ, Sah R, Paniz-Mondolfi A (2020). Should the holy week 2020 be cancelled in Latin America due to the COVID-19 pandemic?. Travel Medicine and Infectious Disease.

[ref-80] Rodriguez-Morales AJ, Sánchez-Duque JA, Hernández Botero S, Pérez-Díaz CE, Villamil-Gómez WE, Méndez CA, Verbanaz S, Cimerman S, Rodriguez-Enciso HD, Escalera-Antezana JP, Balbin-Ramon GJ, Arteaga-Livias FK, Cvetkovic-Vega A, Orduna T, Savio-Larrea E, Paniz-Mondolfi A (2020c). Preparación y control de la enfermedad por coronavirus 2019 (COVID-19) en América Latina. Acta Medica Peruana.

[ref-81] Rosa SGV, Santos WC (2020). Clinical trials on drug repositioning for COVID-19 treatment. Revista Panamericana de Salud Publica-Pan American Journal of Public Health.

[ref-82] Rosales-Mendoza S (2020). Will plant-made biopharmaceuticals play a role in the fight against COVID-19?. Expert Opinion on Biological Therapy: Null–Null.

[ref-83] Saavedra-Velasco M, Chiara-Chilet C, Pichardo-Rodriguez R, Grandez-Urbina A, Inga-Berrospi F (2020). Coinfección entre dengue y COVID-19: Necesidad de abordaje en zonas endémicas. Revista de la Facultad de Ciencias Médicas de Córdoba.

[ref-84] Sabino-Silva R, Jardim ACG, Siqueira WL (2020). Coronavirus COVID-19 impacts to dentistry and potential salivary diagnosis. Clinical Oral Investigations.

[ref-85] Sagulenko P, Puller V, Neher RA (2018). TreeTime: maximum-likelihood phylodynamic analysis. Virus Evolution.

[ref-86] Sah R, Rodriguez-Morales AJ, Jha R, Chu DKW, Gu H, Peiris M, Bastola A, Lal BK, Ojha HC, Rabaan AA, Zambrano LI, Costello A, Morita K, Pandey BD, Poon LLM (2020). Complete genome sequence of a 2019 novel coronavirus (SARS-COV-2) strain isolated in Nepal. Microbiology Resource Announcements.

[ref-87] Sawano T, Ozaki A, Rodriguez-Morales AJ, Tanimoto T, Sah R (2020). Limiting spread of COVID-19 from cruise ships: lessons to be learnt from Japan. QJM: An International Journal of Medicine.

[ref-88] Serafin MB, Bottega A, Foletto VS, Da Rosa TF, Hörner A, Hörner R (2020). Drug repositioning an alternative for the treatment of coronavirus COVID-19. International Journal of Antimicrobial Agents.

[ref-89] Sifuentes-Rodriguez E, Palacios-Reyes D (2020). COVID-19: The outbreak caused by a new coronavirus. Boletin Medico del Hospital Infantil de Mexico.

[ref-90] Siordia JA (2020). Epidemiology and clinical features of COVID-19: a review of current literature. Journal of Clinical Virology.

[ref-91] Stern D, López-Olmedo N, Pérez-Ferrer C, González-Morales R, Canto-Osorio F, Barrientos-Gutiérrez T (2020). Revisión rápida del uso de cubrebocas quirúrgicos en ámbito comunitario e infecciones respiratorias agudas. Salud Pública de México.

[ref-92] Torales J, Higgins M, Castaldelli-Maia JM, Ventriglio A (2020). The outbreak of COVID-19 coronavirus and its impact on global mental health. International Journal of Social Psychiatry.

[ref-93] Torres I, Sacoto F (2020). Localising an asset-based COVID-19 response in Ecuador. Lancet.

[ref-94] Trujillo A (2020). Response to Wen and Li. anesthesia procedure of emergency operation for patients with suspected or confirmed COVID-19 (DOI: 10.1089/sur.2020.040). Surg Infect, (Larchmt).

[ref-95] Van Doremalen N, Bushmaker T, Morris DH, Holbrook MG, Gamble A, Williamson BN, Tamin A, Harcourt JL, Thornburg NJ, Gerber SI, Lloyd-Smith JO, De Wit E, Munster VJ (2020). Aerosol and surface stability of SARS-CoV-2 as compared with SARS-CoV-1. New England Journal of Medicine.

[ref-96] Vega-Vega O, Arvizu-Hernández M, Domínguez-Cherit JG, Sierra-Madero J, Correa-Rotter R (2020). Prevención y control de la infección por coronavirus SARS-CoV-2 (Covid-19) en unidades de hemodiálisis. Salud Pública de México.

[ref-97] Wu D, Wu T, Liu Q, Yang Z (2020). The SARS-CoV-2 outbreak: What we know. International Journal of Infectious Diseases.

[ref-98] Xu B, Gutierrez B, Mekaru S, Sewalk K, Goodwin L, Loskill A, Cohn EL, Hswen Y, Hill SC, Cobo MM, Zarebski AE, Li S, Wu CH, Hulland E, Morgan JD, Wang L, O’Brien K, Scarpino SV, Brownstein JS, Pybus OG, Pigott DM, Kraemer MUG (2020). Epidemiological data from the COVID-19 outbreak, real-time case information. Scientific Data.

[ref-99] Zambrano LI, Fuentes-Barahona IC, Bejarano-Torres DA, Bustillo C, Gonzales G, Vallecillo-Chinchilla G, Sanchez-Martínez FE, Valle-Reconco JA, Sierra M, Bonilla-Aldana DK, Cardona-Ospina JA, Rodríguez-Morales AJ (2020). A pregnant woman with COVID-19 in Central America. Travel Medicine and Infectious Disease.

[ref-100] Zhu N, Zhang D, Wang W, Li X, Yang B, Song J, Zhao X, Huang B, Shi W, Lu R, Niu P, Zhan F, Ma X, Wang D, Xu W, Wu G, Gao GF, Tan W (2020). A novel coronavirus from patients with pneumonia in China, 2019. New England Journal of Medicine.

